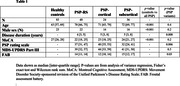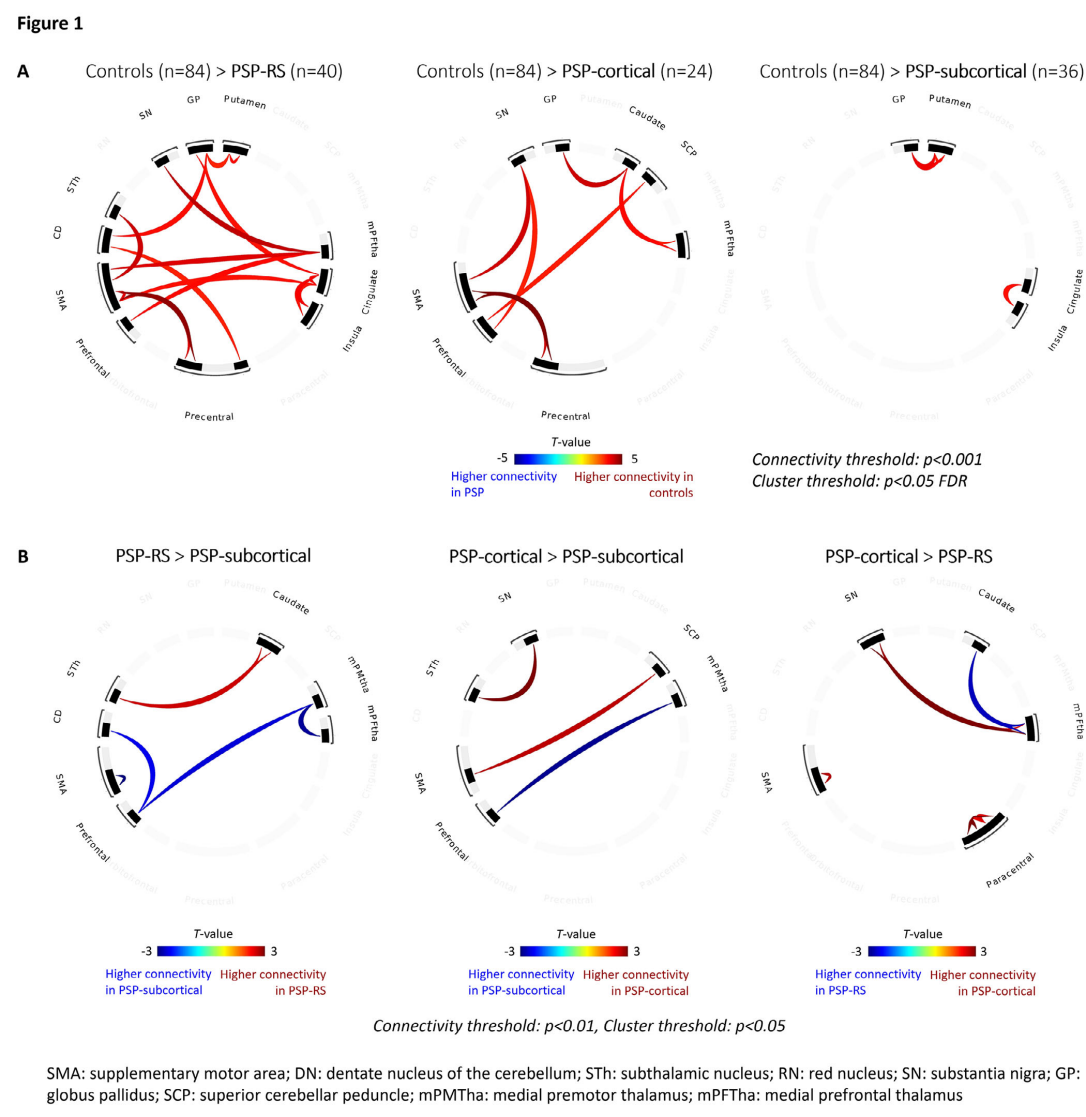# Functional connectivity patterns among clinical variants of progressive supranuclear palsy

**DOI:** 10.1002/alz.085783

**Published:** 2025-01-09

**Authors:** Irene Sintini, Farwa Ali, Yehkyoung C. Stephens, Heather M. Clark, Julie A.G. Stierwalt, Keith A. Josephs, Jennifer L. Whitwell

**Affiliations:** ^1^ Mayo Clinic, Rochester, MN USA

## Abstract

**Background:**

Progressive supranuclear palsy (PSP) can present with different clinical variants which show distinct, but partially overlapping, patterns of neurodegeneration and tau deposition in a PSP network of regions, including cerebellar dentate, superior cerebellar peduncle, midbrain, thalamus, basal ganglia, and frontal lobe. We sought to determine whether disruptions in functional connectivity within this PSP network measured using resting‐state functional MRI (rs‐fMRI) differed between PSP‐Richardson’s syndrome and the cortical and subcortical variants of PSP.

**Method:**

Structural MRI and rs‐fMRI scans were collected for 40 PSP‐RS, 24 PSP‐cortical (12 speech and language; 10 corticobasal syndrome; 2 frontal) and 36 PSP‐subcortical (18 parkinsonism; 11 progressive gait freezing; 6 postural instability; 1 oculomotor) participants who met the Movement Disorder Society PSP clinical criteria (Table 1). Ninety‐six participants underwent flortaucipir‐PET scans. MRIs were processed using CONN Toolbox. Functional connectivity between seeds placed throughout the PSP network was compared between each PSP group and 83 healthy controls, and between the PSP groups, covarying for age and sex.

**Results:**

Connectivity was reduced throughout the network in PSP‐RS compared to controls (Figure 1A), involving cerebellar dentate, midbrain nuclei, subthalamic nuclei, basal ganglia, thalamus, and frontal regions. Frontal regions showed reduced connectivity to other regions in the network in PSP‐cortical, including substantia nigra and superior cerebellar peduncle. Disruptions in connectivity in PSP‐subcortical were less pronounced, with the strongest disruption between the pallidum and putamen. When PSP groups were compared to each other (Figure 1B), PSP‐RS had lower connectivity from the thalamus and cerebellar dentate to the cortex compared to PSP‐subcortical and from the thalamus to substantia nigra than PSP‐cortical. PSP‐subcortical had lower connectivity from the subthalamic nucleus to caudate than PSP‐RS and from the subthalamic nucleus to substantia nigra than PSP‐cortical and higher connectivity from the thalamus to the prefrontal cortex than both the other variants. Voxel‐based analyses replicated these patterns. There was moderate evidence that tau uptake in the PSP network regions influenced connectivity between these regions in PSP.

**Conclusions:**

Patterns of disrupted functional connectivity revealed both variant‐specific and shared disease pathways among PSP variants, providing insight into the disease heterogeneity beyond patterns of atrophy.